# Wernicke’s Encephalopathy in a Young Non-alcoholic Woman Secondary to Prolonged Reduced Oral Intake: A Case Report

**DOI:** 10.7759/cureus.114674

**Published:** 2026-08-17

**Authors:** Harmanpreet Jabbal, Muqeet Enver, Duaa Masood Ahmed

**Affiliations:** 1 Geriatrics, Walsall Manor Hospital, Walsall, GBR; 2 Diabetes and Endocrinology, Walsall Manor Hospital, Walsall, GBR; 3 Nephrology, Russells Hall Hospital, Dudley, GBR

**Keywords:** acute confusion, bilateral ophthalmoplegia, gait ataxia, malnutrition, non-alcoholic, non-alcoholic wernicke’s encephalopathy, wernicke’s encephalopathy (we)

## Abstract

Wernicke's encephalopathy (WE) is an acute neurological disorder resulting from thiamine deficiency and is classically associated with chronic alcohol misuse. However, it may also occur in non-alcoholic patients with prolonged malnutrition, persistent vomiting, or impaired nutritional intake, making diagnosis particularly challenging when the classical triad of ophthalmoplegia, ataxia, and altered mental status is incomplete or develops gradually. Delayed recognition can result in irreversible neurological injury, whereas early treatment with thiamine is associated with promising outcomes. We report the case of a 26-year-old woman who presented with persistent vomiting, significant weight loss, and poor oral intake. During her admission, she developed acute binocular diplopia, bilateral ophthalmoplegia, gait ataxia, confusion, and short-term memory impairment. She fulfilled all four Caine criteria for WE, demonstrating dietary deficiency, oculomotor abnormalities, cerebellar dysfunction, and altered mental state/memory impairment. Initial neuroimaging raised concern for pituitary pathology; however, subsequent endocrine investigations and vascular imaging excluded pituitary apoplexy and adrenal insufficiency. Nerve conduction studies and cerebrospinal fluid analysis were unremarkable, excluding peripheral demyelinating disease. Given the characteristic clinical presentation in the setting of prolonged nutritional deficiency, a diagnosis of WE was made, and treatment with intravenous thiamine (Pabrinex) was initiated. The patient demonstrated rapid neurological improvement, with resolution of confusion, marked recovery of ocular signs and ataxia, and improvement in Montreal Cognitive Assessment score from 16/30 to 28/30 before discharge. This case highlights the importance of maintaining a high index of suspicion for WE in non-alcoholic patients presenting with prolonged vomiting and malnutrition. It also emphasises the utility of the Caine criteria in supporting the diagnosis when the classical triad is not initially apparent. Although initial neuroimaging raised concerns regarding pituitary pathology, subsequent investigations excluded alternative diagnoses, reinforcing that imaging findings should not distract from the clinical diagnosis of WE. Prompt empirical thiamine replacement remains essential to prevent permanent neurological damage and achieve excellent clinical recovery.

## Introduction

Wernicke’s encephalopathy (WE) is an acute neurological condition resulting from thiamine (vitamin B1) deficiency requiring prompt recognition and treatment. Thiamine is an essential cofactor in cerebral glucose metabolism, and deficiency leads to impaired neuronal energy production, oxidative stress, and selective injury to metabolically active regions of the brain, including the mammillary bodies, medial thalami, periaqueductal grey matter, and cerebellum [1,2]. If left untreated, WE may progress to irreversible neurological sequelae, including Korsakoff syndrome, or result in death [1,3]. It is commonly associated with chronic alcohol misuse and less recognised in patients presenting with conditions causing malnutrition or prolonged nutritional deficiencies [1,4]. 

The diagnosis remains challenging because the classical triad of encephalopathy, ophthalmoplegia, and gait ataxia is present in only a minority of patients, particularly in non-alcoholic populations, resulting in frequent delays in diagnosis and treatment [3,5]. Furthermore, neuroimaging findings may be absent or atypical, reinforcing that WE is primarily a clinical diagnosis, and that treatment with intravenous thiamine should not be delayed when clinical suspicion exists [2,6]. 

We report the case of a previously healthy 26-year-old non-alcoholic woman who developed WE following prolonged poor oral intake and persistent vomiting associated with significant weight loss. The diagnosis was initially complicated by incidental pituitary imaging abnormalities, but the patient demonstrated marked neurological recovery following prompt intravenous thiamine replacement. This case highlights the importance of considering WE in any nutritionally depleted patient presenting with acute neurological symptoms, even in the absence of alcohol misuse or more commonly recognised risk factors for thiamine deficiency.

## Case presentation

A 26-year-old woman presented to the emergency department with epigastric pain accompanied by multiple episodes of vomiting. She reported a two-month history of persistent nausea, markedly reduced oral intake, and an inability to tolerate both food and fluids. Over the preceding three months, she had experienced an unintentional weight loss of approximately 19 kg (3 stone), which she attributed to loss of appetite and recurrent vomiting. Her BMI at admission was 25.8 kg/m² (77 kg, 173 cm), which falls within the overweight range; however, she had experienced substantial unintentional weight loss, representing around 20% of her body weight and indicating significant recent nutritional depletion despite a non-malnourished BMI.

Her past medical history included chronic fatigue syndrome, fibromyalgia, inflammatory arthritis, autism spectrum disorder, depression, and known cholelithiasis. Given her psychiatric comorbidities and subsequent history of prolonged reduced oral intake and vomiting, an eating disorder or a psychogenic contribution to her nutritional decline was considered but not supported by the clinical history. Her regular medications comprised omeprazole and citalopram. She had no known drug allergies. There was no family history of neurological or autoimmune disease. She was a lifelong non-smoker, consumed alcohol only occasionally, and denied recreational drug use. 

The patient was initially admitted under the general surgical team for investigation of possible hepatobiliary or pancreatic pathology. Contrast-enhanced computed tomography of the abdomen and pelvis demonstrated a large gallstone without evidence of acute cholecystitis, pancreatitis, appendicitis, or other acute intra-abdominal pathology. Blood investigation results obtained during the admission are shown in Table 1. 

**Table 1:** Blood investigation results

Parameter	Patient value	Reference range
Haemoglobin	125 g/L	115–165 g/L
White cell count	5.90 x 10^9^/L	4.0–11.0 x 10^9^/L
Neutrophils	2.34 x 10^9^/L	2.0–7.5 x 10^9^/L
Lymphocytes	3.00 x 10^9^/L	1.5–4.5 x 10^9^/L
C-reactive protein	13 mg/L	0–5 mg/L
Alanine aminotransferase	78 IU/L	0–55 IU/L
Alkaline phosphatase	62 IU/L	30–130 IU/L
Bilirubin	11 μmol/L	5–26 μmol/L
Adjusted calcium	2.46 mmol/L	2.20–2.60 mmol/L
Vitamin B12	>128 pmol/L	51–128 pmol/L
Folate	6.5 μg/L	3.1–20.5 μg/L
Vitamin B1 (thiamine)	114 nmol/L	66.5–200 nmol/L
Zinc	13.6 μmol/L	11.0–24.0 μmol/L
Magnesium	0.79	0.7-1.0 mmol/L
Ganglioside antibodies	Negative	–
Autoimmune encephalitis screen	Negative	–
Prolactin	255 mIU/L	63–561 mIU/L
Luteinising hormone	<0.1 IU/L	–
Follicle-stimulating hormone	0.6 IU/L	–
Insulin-like growth factor 1	7.4 nmol/L	10.2–35.1 nmol/L
Thyroid-stimulating hormone	1.39 mIU/L	0.35-4.94 mIU/L
Free T4	15.9 pmol/L	9.0-19.0 pmol/L
9am cortisol	330 nmol/L	102 - 535 nmol/L
Adrenocorticotrophic hormone	6 ng/L	6 - 51 ng/L

During her admission, she developed acute binocular diplopia associated with progressive gait instability and ataxia, resulting in a fall. Before admission, she was fully independent in mobility and activities of daily living; however, she subsequently required assistance with ambulation. Ophthalmological assessment demonstrated bilateral ophthalmoplegia with binocular diplopia on lateral gaze; impaired horizontal eye movements affecting both abduction and adduction; vertical gaze restriction with upbeat nystagmus on upward gaze; and preserved visual acuity and visual fields. 

Neurological examination revealed normal muscle bulk and tone with preserved power (Medical Research Council grade 5/5) in all four limbs. The sensory examination was unremarkable. Deep tendon reflexes were preserved throughout, except for absent bilateral ankle jerks. Plantar responses were downgoing bilaterally. Marked truncal and gait ataxia was present, preventing independent mobilisation. The absent ankle jerks raised the possibility of peripheral involvement related to thiamine deficiency; however, subsequent nerve conduction studies, including sensory studies and F-wave responses, were entirely normal, with no electrophysiological evidence of peripheral neuropathy. This isolated clinical finding therefore remained unexplained.

**Figure 1:**
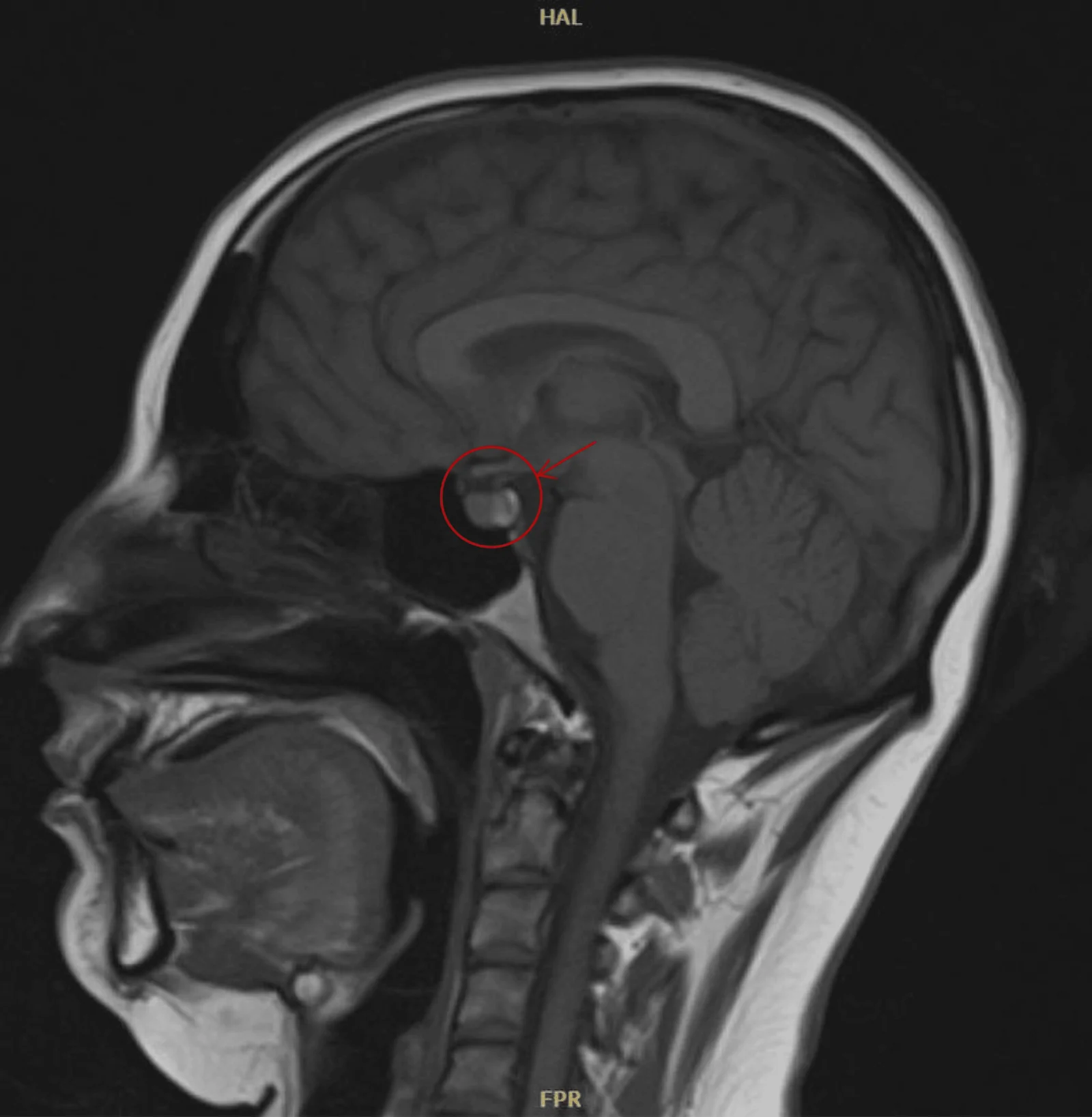
Magnetic resonance imaging findings Magnetic resonance imaging of the head demonstrates a lesion with high signal intensity on T1-weighted imaging and low signal intensity on T2-weighted and fluid-attenuated inversion recovery imaging, without restricted diffusion. The imaging differential diagnoses include calcification and chronic haemorrhage.

Magnetic resonance imaging of the brain and pituitary gland with gadolinium contrast demonstrated signal abnormality within the posterior pituitary, initially raising the possibility of calcification or previous haemorrhage, and pituitary apoplexy was considered (Figure 1 - see legend). At the time of imaging, she had developed bilateral ophthalmoplegia and gait ataxia but had no acute headache or visual field disturbance, and there were no other clinical features specifically suggestive of pituitary apoplexy or cavernous sinus involvement. In retrospect, the posterior pituitary signal abnormality may have represented the normal T1-hyperintense posterior pituitary “bright spot”, reflecting physiological neurohypophyseal vasopressin and neurophysin storage. Nevertheless, given the initial radiological concern and her evolving neurological presentation, further endocrine investigations, CT cerebral angiography, and neurosurgical consultation were undertaken. CT cerebral angiography demonstrated normal intracranial vasculature with no significant abnormality involving the pituitary region, and subsequent endocrine investigations did not support pituitary apoplexy or adrenal insufficiency.

A short Synacthen test demonstrated an adequate adrenal response, with serum cortisol concentrations increasing from 152 nmol/L at baseline (typically 140-500 nmol/L) to 684 nmol/L at 30 minutes (adequate response ≥450-500 nmol/L) and 740 nmol/L at 60 minutes (adequate response ≥500-550 nmol/L), effectively excluding adrenal insufficiency. Consequently, corticosteroid therapy was discontinued. 

During admission, the patient's neurological condition evolved further, with the development of acute confusion, impaired short-term memory, repetitive questioning, and reduced spontaneous speech. Cognitive assessment using the Montreal Cognitive Assessment (MoCA) yielded a score of 16/30, consistent with moderate cognitive impairment. 

Given the combination of ophthalmoplegia, gait ataxia, cognitive dysfunction, and a history of prolonged malnutrition secondary to persistent vomiting, WE was considered the leading diagnosis. Intravenous Pabrinex (high-potency vitamin B and C complex) was commenced - two pairs, three times per day, for three days, followed by maintenance oral thiamine and multivitamin supplementation. Additional investigations, including lumbar puncture and nerve conduction studies, were performed to exclude alternative neurological diagnoses. 

Nerve conduction studies demonstrated normal motor and sensory conduction parameters, with preserved distal latencies, amplitudes, conduction velocities, and F-wave responses, providing no electrophysiological evidence of peripheral neuropathy or demyelinating disease (Table 2). Cerebrospinal fluid analysis was unremarkable (Table 3). 

**Table 2:** Nerve conduction study findings CMAP: compound muscle action potential, SNAP: sensory nerve action potential

Nerve/study	Electrophysiological findings
Right median motor nerve	Normal distal latency, CMAP amplitude, and conduction velocity
Bilateral peroneal motor nerves	Normal distal latency and conduction velocity with preserved CMAP amplitudes, no evidence of conduction block
Bilateral tibial motor nerves	Normal distal latency and CMAP amplitudes
Right ulnar motor nerve	Normal distal latency, CMAP amplitudes, and conduction velocities across the elbow
Bilateral superficial peroneal sensory nerves	Normal SNAP amplitudes, distal latencies, and sensory conduction velocities
Bilateral sural sensory nerves	Normal SNAP amplitudes, distal latencies, and sensory conduction velocities
Right median, right ulnar, and right radial sensory nerves	Normal SNAP amplitudes, distal latencies, and sensory conduction velocities
Tibial F-wave study	Normal minimum F-wave latencies bilaterally
Overall interpretation	Normal motor and sensory nerve conduction studies with preserved F-wave responses, indicating no electrophysiological evidence of a peripheral neuropathy or demyelinating process.

**Table 3:** Cerebrospinal fluid analysis findings

Parameter	Patient value	Reference range
Appearance	Clear colourless fluid	Clear
Total Leucocyte count	0 cells/μL	0–5 cells/μL
Cerebrospinal fluid protein	0.80 g/L	0.15–0.45 g/L
Cerebrospinal fluid glucose	3.5 mmol/L	2.2–3.9 mmol/L
Gram stain/culture	No organisms seen	–
Cerebrospinal fluid encephalitis screen	Negative	–

Following the initiation of thiamine replacement therapy, the patient demonstrated rapid clinical improvement. Her confusion, short-term memory impairment, and gait ataxia improved substantially, with her MoCA score increasing from 16/30 to 28/30 prior to discharge. Ocular findings, including upbeat nystagmus, ophthalmoplegia, and diplopia, also showed marked improvement. By the time of discharge, she had regained the ability to mobilise independently using a walking frame. She was discharged with ongoing oral thiamine supplementation and outpatient neurology and ophthalmology follow-up. 

## Discussion

WE is an acute neurological deficiency associated with vitamin B1 (thiamine) deficiency. WE is classically seen in patients with chronic alcohol abuse; however, an increased number of cases have been identified in patients with prolonged malnutrition resulting from eating disorders, bariatric surgery, malignancy, gastrointestinal disorders, and hyperemesis gravidarum [1,2]. Although there is growing recognition, clinicians often miss this diagnosis in non-alcoholic populations due to a lower index of suspicion [1,3]. 

Thiamine is an essential water-soluble vitamin that functions as a co-factor for several enzymes involved in cerebral glucose metabolism, including pyruvate dehydrogenase, α-ketoglutarate dehydrogenase, and transketolase. Thiamine deficiency results in impaired aerobic metabolism, reduced ATP production, oxidative stress, neuronal injury, and cytotoxic oedema. Brain regions with high metabolic requirements, including the medial thalami, mammillary bodies, periaqueductal grey matter, and cerebellar vermis, are particularly susceptible to injury, explaining the characteristic ocular abnormalities, gait ataxia, and cognitive impairment seen in WE [1,6]. 

Most reported cases of non-alcoholic WE occur following bariatric surgery, hyperemesis gravidarum, gastrointestinal malabsorption, or malignancy, none of which were present in our patient. Instead, she had experienced two months of markedly reduced appetite and oral intake, accompanied by persistent vomiting and approximately 19 kg of weight loss. Despite the absence of biochemical evidence of thiamine deficiency, the combination of substantial recent nutritional depletion and subsequent characteristic neurological features supported a clinical diagnosis of WE. This case therefore highlights that prolonged inadequate nutritional intake alone can precipitate WE in previously healthy adults. Given that total body thiamine stores are limited and may become depleted within approximately two to three weeks without adequate intake, prolonged vomiting and starvation represent important but sometimes overlooked risk factors [1,2,7].

This case presented a significant diagnostic challenge due to both atypical neuroimaging findings and a normal serum thiamine concentration. The initial MRI scan demonstrated posterior pituitary signal abnormalities, raising concern for pituitary apoplexy and prompting further endocrine investigations, CT angiography, neurosurgical consultation, and initiation of corticosteroid therapy. The endocrine abnormalities were also considered in the context of her significant recent weight loss and nutritional depletion. Markedly suppressed LH and FSH and a low IGF-1, with preserved thyroid function and normal prolactin, may reflect functional suppression of the hypothalamic-pituitary axes secondary to negative energy balance. These findings provide a plausible explanation for the endocrine abnormalities without necessarily indicating structural pituitary disease. The posterior pituitary imaging abnormality was ultimately not considered to represent clinically significant structural pathology but contributed to diagnostic uncertainty at a time when the patient's neurological presentation was evolving. Importantly, the patient's serum thiamine concentration was within the normal reference range; however, circulating thiamine levels do not necessarily reflect intracellular or cerebral thiamine availability, and no serum threshold reliably excludes WE. Serum concentrations may therefore be normal despite clinically significant tissue or functional deficiency, meaning that a normal result should not override a compatible clinical presentation. In this case, the subsequent development of bilateral ophthalmoplegia, gait ataxia, confusion, and memory impairment in the context of prolonged vomiting, poor oral intake, and substantial recent weight loss fulfilled all four Caine criteria for WE. The diagnosis was therefore made clinically, supported by the marked neurological response to intravenous thiamine, despite the absence of biochemical confirmation. This highlights the importance of integrating nutritional history and evolving neurological findings rather than relying on serum thiamine levels or atypical imaging findings in isolation [2,6].

Our patient eventually developed all three components of the classical triad of WE: ophthalmoplegia with gaze-evoked nystagmus, gait ataxia resulting in falls, and encephalopathy characterised by confusion and short-term memory impairment. However, the complete classical triad is present in only a minority of patients, and the Caine criteria provide a more sensitive clinical approach to diagnosis, with WE supported by the presence of at least two of four features: dietary deficiency, oculomotor abnormalities, cerebellar dysfunction, and altered mental state or mild memory impairment [8]. In our patient, all four criteria were met, with significant recent weight loss and poor oral intake indicating dietary deficiency, bilateral ophthalmoplegia representing oculomotor dysfunction, gait ataxia indicating cerebellar involvement, and confusion with short-term memory impairment demonstrating altered mental state and cognitive dysfunction. Harper et al. reported that the classic triad is identified in only around 16% of patients at presentation, contributing substantially to missed diagnoses [3]. Consequently, current European Federation of Neurological Societies (EFNS) guidelines recommend maintaining a low threshold for empirical treatment in any patient with nutritional deficiency who develops otherwise unexplained neurological symptoms [2]. 

MRI has relatively low sensitivity but high specificity for WE; imaging may be normal or demonstrate atypical findings, particularly in non-alcoholic patients [6,9]. In our patient, the MRI did not demonstrate the characteristic findings (i.e., symmetrical T2-weighted and FLAIR hyperintensities involving the medial thalami, mammillary bodies, tectal plate, and periaqueductal grey matter); however, it revealed a posterior pituitary signal abnormality that initially raised concern for pituitary pathology. This case demonstrates that neuroimaging should not delay the initiation of treatment when clinical suspicion for WE exists.

Furthermore, nerve conduction studies and lumbar puncture were performed to investigate alternative acute neurological diagnoses. Both were unremarkable, making peripheral demyelinating disease and central nervous system infection or inflammation less likely. The isolated finding of absent bilateral ankle jerks raised the possibility of peripheral involvement related to thiamine deficiency; however, normal nerve conduction studies provided no electrophysiological evidence of peripheral neuropathy. Although these investigations are often appropriate in atypical presentations, they should not postpone administration of intravenous thiamine, which is inexpensive, well tolerated, and carries minimal risk [2,10]. 

Prompt recognition of WE is essential because delayed treatment may result in irreversible neurological injury, progression to Korsakoff syndrome, or death [1,2,5]. Consequently, treatment should be initiated immediately whenever clinical suspicion exists, without waiting for confirmatory investigations [2,10]. 

This case highlights multiple clinical learning points. First, WE should not be regarded solely as a complication of chronic alcohol misuse and should remain within the differential diagnosis in patients with acute neurological symptoms and evidence of nutritional deficiency. Second, significant nutritional depletion can occur despite a preserved or elevated BMI, particularly following prolonged vomiting and inadequate oral intake, and may be sufficient to precipitate WE in the absence of traditional risk factors or underlying malabsorptive disorders. Third, incidental neuroimaging findings may create diagnostic uncertainty and should not detract from careful clinical assessment. Finally, this case illustrates the remarkable neurological recovery that is possible when intravenous thiamine is administered promptly, emphasising the importance of maintaining a high index of suspicion for WE in any patient presenting with acute ophthalmoplegia, ataxia, or cognitive impairment. 

## Conclusions

This case illustrates that WE can develop despite a preserved or elevated BMI and in the absence of alcohol misuse, particularly following substantial recent weight loss and prolonged vomiting. The presence of neurological features fulfilling the Caine criteria should prompt consideration of thiamine deficiency, even when alternative diagnoses are suggested by initial investigations. Recognition of the clinical pattern and timely empirical thiamine replacement are crucial, as early treatment can lead to marked neurological recovery and minimise the risk of permanent sequelae.

## References

[1] Sechi G, Serra A (2007). Wernicke's encephalopathy: new clinical settings and recent advances in diagnosis and management. Lancet Neurol.

[2] Galvin R, Bråthen G, Ivashynka A, Hillbom M, Tanasescu R, Leone MA (2010). EFNS guidelines for diagnosis, therapy and prevention of Wernicke encephalopathy. Eur J Neurol.

[3] Harper CG, Giles M, Finlay-Jones R (1986). Clinical signs in the Wernicke-Korsakoff complex: a retrospective analysis of 131 cases diagnosed at necropsy. J Neurol Neurosurg Psychiatry.

[4] Oudman E, Wijnia JW, Oey MJ, van Dam M, Postma A (2021). Wernicke-Korsakoff syndrome despite no alcohol abuse: a summary of systematic reports. J Neurol Sci.

[5] Isenberg-Grzeda E, Kutner HE, Nicolson SE (2012). Wernicke-Korsakoff-syndrome: under-recognized and under-treated. Psychosomatics.

[6] Manzo G, De Gennaro A, Cozzolino A, Serino A, Fenza G, Manto A (2014). MR imaging findings in alcoholic and nonalcoholic acute Wernicke's encephalopathy: a review. Biomed Res Int.

[7] Thomson AD, Marshall EJ (2006). The natural history and pathophysiology of Wernicke's encephalopathy and Korsakoff's psychosis. Alcohol Alcohol.

[8] Caine D, Halliday GM, Kril JJ, Harper CG (1997). Operational criteria for the classification of chronic alcoholics: identification of Wernicke's encephalopathy. J Neurol Neurosurg Psychiatry.

[9] Antunez E, Estruch R, Cardenal C, Nicolas JM, Fernandez-Sola J, Urbano-Marquez A (1998). Usefulness of CT and MR imaging in the diagnosis of acute Wernicke's encephalopathy. AJR Am J Roentgenol.

[10] Thomson AD, Cook CC, Touquet R, Henry JA (2002). The Royal College of Physicians report on alcohol: guidelines for managing Wernicke's encephalopathy in the accident and Emergency Department. Alcohol Alcohol.

